# Alcohol exposure alters the diversity and composition of oral microbiome

**DOI:** 10.3389/fcimb.2025.1561055

**Published:** 2025-04-07

**Authors:** Zirui Zhao, Jiaxin Li, Juan Liu, Xiao Zhang, Yusen Qie, Yutong Sun, Na Liu, Qing Liu

**Affiliations:** ^1^ Hebei Key Laboratory of Stomatology/Hebei Technology Innovation Center of Oral Health, School and Hospital of Stomatology, Hebei Medical University, Shijiazhuang, Hebei, China; ^2^ Department of Preventive Dentistry, School and Hospital of Stomatology, Hebei Medical University, Shijiazhuang, Hebei, China

**Keywords:** alcohol exposure, microbiome, oral health, bacteria, 16S rRNA amplicon

## Abstract

**Introduction:**

Alcohol exposure has been shown to have complex, and sometimes paradoxical, associations with various serious diseases. Currently, there is no knowledge about the effects of alcohol exposure on the dynamics of oral microbial communities.

**Objective:**

The study aims to investigate the effects of chronic alcohol consumption on the diversity and composition dynamics of the rat oral microbiota using 16S rRNA gene amplicon sequencing.

**Methods:**

In our study, there were 2 groups, including a control group (C group) and an alcohol group (A group), with 10 rats in every group. For ten weeks, rats in the A group were treated with alcohol intragastrically every day, whereas rats in the C group got water. After 10 weeks, serum levels of alanine aminotransferase (ALT) and aspartate aminotransferase (AST) were measured. Oral swabs were taken from both groups, and total DNA was extracted for high-throughput sequencing of the 16S rRNA gene.

**Results:**

According to the results obtained from our study, significant differences were observed in the relative abundances of microbial communities. Alpha diversity measures were statistically significantly higher (P < 0.05) in the A group compared to the C group. At the genus level, alcohol exposure altered the relative abundance of several microbes, including increased relative abundance of *unidentified_Chloroplast, Acinetobacter, Vibrio, Romboutsia, Pseudoalteromonas, Aeromonas, Ralstonia, Turicibacter, Shewanella*, and *Bacteroides*. Conversely, *Haemophilus* and *Streptococcus* were significantly less abundant in the A group.

**Conclusion:**

Alcohol exposure was associated with the diversity and composition of the oral microbiome. These findings contribute to our understanding of the potential role of oral bacteria in alcohol-related oral and systemic diseases, providing foundational work for future prevention and intervention studies.

## 1 Introduction

The oral cavity has a diverse microbiome, ranking as the second most complex ecosystem after the gut. This microbiome comprises a multitude of microorganisms, including bacteria, archaea, fungi, viruses, and protozoa, with over 700 distinct species identified (Santacroce et al., 2023). The oral microbiome forms a unique, homeostatic community that maintains a dynamic equilibrium with the host’s immune system (Lee et al., 2021). Dysbiosis, characterized by alterations in the presence or absence of specific microbial species or a reduction in overall microbial diversity (Radaic and Kapila, 2021), disrupts this delicate balance and is essential for preserving human health. The homeostasis of the oral microbiome is pivotal in the context of numerous diseases.

Alcohol exposure is associated with a myriad of immediate and chronic health issues (Graham et al., 2011), accounting for over 170,000 annual fatalities in the United States (Esser et al., 2024). Health outcomes associated with alcohol consumption include cardiovascular diseases (Roerecke, 2021), nutritional deficiencies (Zahr et al., 2016), various forms of cancer (Stornetta et al., 2018), premature aging (Luo et al., 2020), and oral health disorders (Pinto et al., 2024). Acetaldehyde, a byproduct of alcohol metabolism, and ethanol itself are both toxic to human cells (Khalatbari et al., 2023). Moreover, the use of alcohol and tobacco exerts a synergistic influence on oral cancer risk (Mello et al., 2019), potentially due to ethanol’s ability to enhance the absorption of carcinogens by increasing the permeability of oral keratinocyte cell membranes, thereby facilitating the uptake of tobacco-derived carcinogens (Rwere et al., 2024).

Alcohol exposure may create an oral environment conducive to the proliferation of pathogenic microorganisms, including bacterial communities and oral phages as well as their interactions, thereby disrupting the structure and function of the oral microbiota (Banar et al., 2024) and increasing the risk of oral diseases such as periodontitis. Notably, while periodontitis itself induces alterations in the oral microbial flora, alcohol exposure introduces further complexity to these microbial community changes.

Ethanol and its metabolites, notably acetaldehyde, have the potential to indirectly alter the oral microenvironment through systemic pathways, including immune modulation and increased oxidative stress (Le Daré et al., 2019). Additionally, alcohol-induced gut dysbiosis may trigger a pro-inflammatory cascade, potentially exacerbating oral dysbiosis (Hu et al., 2023). Intragastric alcohol administration, which circumvents direct oral exposure with ethanol, offers a distinct model to delineate systemic effects of alcohol metabolism from localized oral interactions. To elucidate the interaction between alcohol exposure and oral dysbiosis, we conducted a microbial study within a rat model of alcohol gavage.

In the current study, we employed 16S rRNA gene high-throughput sequencing to evaluate the oral microbiota exposed to prolonged alcohol intake. We provide insights into the relationship between alcohol exposure and the oral microbiota under controlled conditions and enhancing our comprehension of the negative health effects of alcohol exposure.

## 2 Methods

### 2.1 Animals rearing

A total of 20 male Wistar rats (Beijing Huafukang Bioscience & Technology Co., Ltd., Beijing, China), 7-8 weeks old and weighing 190-220g, were used in the study (license number: SCXK (Jing) 2019-0008). In line with the inclusion and exclusion criteria of previous clinical study (Avetisyan et al., 2021), the rats in this study were verified to have intact oral mucosa and were free from systemic infections. The sample size was determined according to existing protocols for mucosal microbiome studies (Pushalkar et al., 2012), ensuring sufficient power to detect clinically relevant microbial changes. The rats were accommodated at the Experimental Animal Public Service Platform of Hebei Medical University, provided with ad libitum access to water and standard rodent chow, and maintained in a controlled environment with a temperature range of 20-25°C and relative humidity of 40-70%. Each cage accommodated two rats. The experimental animals were subjected to a 12/12-hour light-dark cycle, with lights on from 7:00 PM to 7:00 AM. A one-week acclimatization period was observed in the animal facility before the commencement of experiments. The experimental protocol for this study received approval from the Medical Ethics Committee of the Stomatology Hospital at Hebei Medical University (approval code: [2022]001). And the study are reported per ARRIVE guidelines.

### 2.2 Study design

20 rats were randomly assigned to two groups: an alcohol group (A group) and a control group (C group). The A group received a 25% (v/v) alcohol solution at a dosage of 2.5g/kg intragastrically on 6 of 7 days per week, while the C group received an equivalent volume of distilled water intragastrically. In animal studies, alcohol administration regimens spanning 4-12 weeks are commonly employed to recapitulate the adverse effects of chronic alcohol consumption observed in humans (Lamas-Paz et al., 2018). Since a month or longer of drinking of rats is considered chronic administration (D’Souza El-Guindy et al., 2010), we employed a 10 - week intragastric administration of a 25% alcohol solution to rats, mimicking human chronic alcohol consumption.

### 2.3 Biochemical analysis

At the end of the 10th week, blood samples were obtained from the ophthalmic venous plexus, allowed to coagulate at room temperature for 30 minutes, and then centrifuged at 13,000 rpm for 10 minutes. The supernatant serum was carefully aspirated and stored at -80°C until analysis. Aalanine aminotransferase (ALT) and aspartate aminotransferase (AST) levels in serum samples were measured using a fully automatic biochemical analyzer (Mindray, Shenzhen, China), strictly following the manufacturer’s instructions.

### 2.4 Oral swab specimen collection and processing

Sterile oral swabs were used under isoflurane anesthesia to collect samples from the oral cavity, systematically sampling the dorsal surface of the tongue, cheek, palate, molars, ventral surface of the tongue, floor of the mouth, incisors, and vestibular groove. Throughout the sampling procedures, rigorous anti-contamination protocols were implemented, to minimize contamination. The swab samples were stored at −80°C until assayed. Samples were provided to Beijing Novogene Co., Ltd. for DNA extraction and 16s rRNA gene sequencing on the Illumina MiSeq platform.

Microbial DNA from oral swabs was extracted using the magnetic bead soil and fecal genomic DNA extraction kit (Tiangen, Beijing, China), following the manufacturer’s guidelines. The V3-V4 region of the 16S rRNA gene was amplified from the DNA samples using a Bio-rad T100 gradient PCR instrument, adhering to the Earth Microbiome Project protocols. The PCR was conducted with primers 341F (CCTAYGGGRBGCASCAG) and 806R (GGACTACNNGGGTATCTAAT), which incorporate barcodes. PCR products were pooled in equimolar amounts and purified using the GeneJET Gel Extraction Kit (Thermo Scientific, USA). Library construction was performed using the TruSeq DNA PCR-Free Library Preparation Kit (Illumina, USA. PE250 sequencing was carried out utilizing the Illumina MiSeq.

### 2.5 Bioinformatics analysis of 16SrRNA gene sequencing

16S rRNA gene sequencing bioinformatics analysis was performed using QIIME2. FLASH (Version 1.2.11) was employed to assemble raw sequence reads, generating raw data. The assembled Raw Tags were processed with fastp software to produce clean data. After the removal of chimeric sequences and noise reduction, the Amplicon Sequence Variants (ASVs) were annotated with species information based on the Silva 138.1 database.

Alpha diversity indices were calculated using QIIME2 to assess species diversity differences between groups, with significance determined by the Kruskal-Wallis rank sum test. R packages (v4.0.3) were used to plot rarefaction curves, ensuring the sequencing depth was adequate. Beta diversity analysis with QIIME2 compared microbial community compositions across samples and generated a heatmap of the distance matrix. Principal coordinate analysis (PCoA) and Non-Metric Multi-Dimensional Scaling analysis (NMDS) were conducted to visualize sample variation. Anosim, Adonis, and MetaStat analyses were performed using T-tests and Wilcoxon tests with R software (v4.0.3). LEfSe software was applied for LDA Effect Size (LEfSe) analysis (LDA>4), presenting results as bar charts and cladograms. Tax4Fun software (V0.3.1) was utilized to predict the Kyoto Encyclopedia of Genes and Genomes (KEGG) pathway functional profiles of microbial communities based on ASV sequences. The analysis of microbial co-occurrence networks was conducted utilizing Spearman’s rank correlation coefficients, identifying significant associations where the Spearman’s |R| > 0.6 and *P*< 0.05. The network topology was then depicted employing Cytoscape software.

### 2.6 Statistical analysis of the general sample

General sample statistical analysis experimental data were analyzed using SPSS 27 statistical software. Quantitative data were expressed as the mean ± standard deviation (X ± S). A T-test was employed when the data followed a normal distribution with equal variances. Statistical significance was set at a *P*< 0.05.

## 3 Results

### 3.1 Biochemical findings

**Figure 1:**
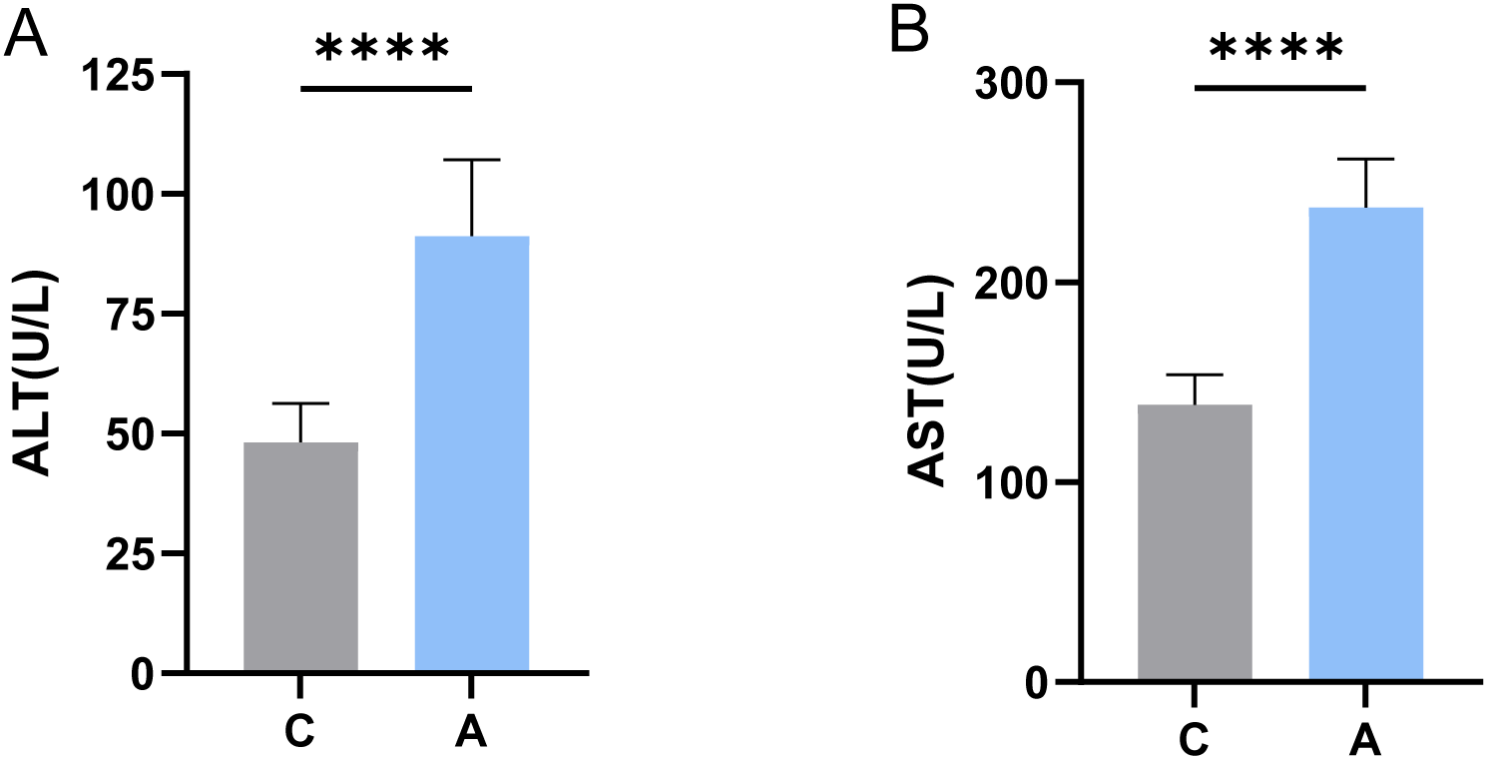
Levels of ALT and AST in serum were determined. **** indicates statistically significant differences compared to the C group (*P* < 0.0001, *independent two-sample t-test*). **(A)** ALT; **(B)** AST.

A statistically significant difference was observed between ALT and AST levels in the A and C groups (*P* < 0.0001) (**Figure 1**).

### 3.2 Sequencing results of 16S amplicons from oral swabs

#### 3.2.1 Alcohol exposure affects microbial community structure

**Figure 2:**
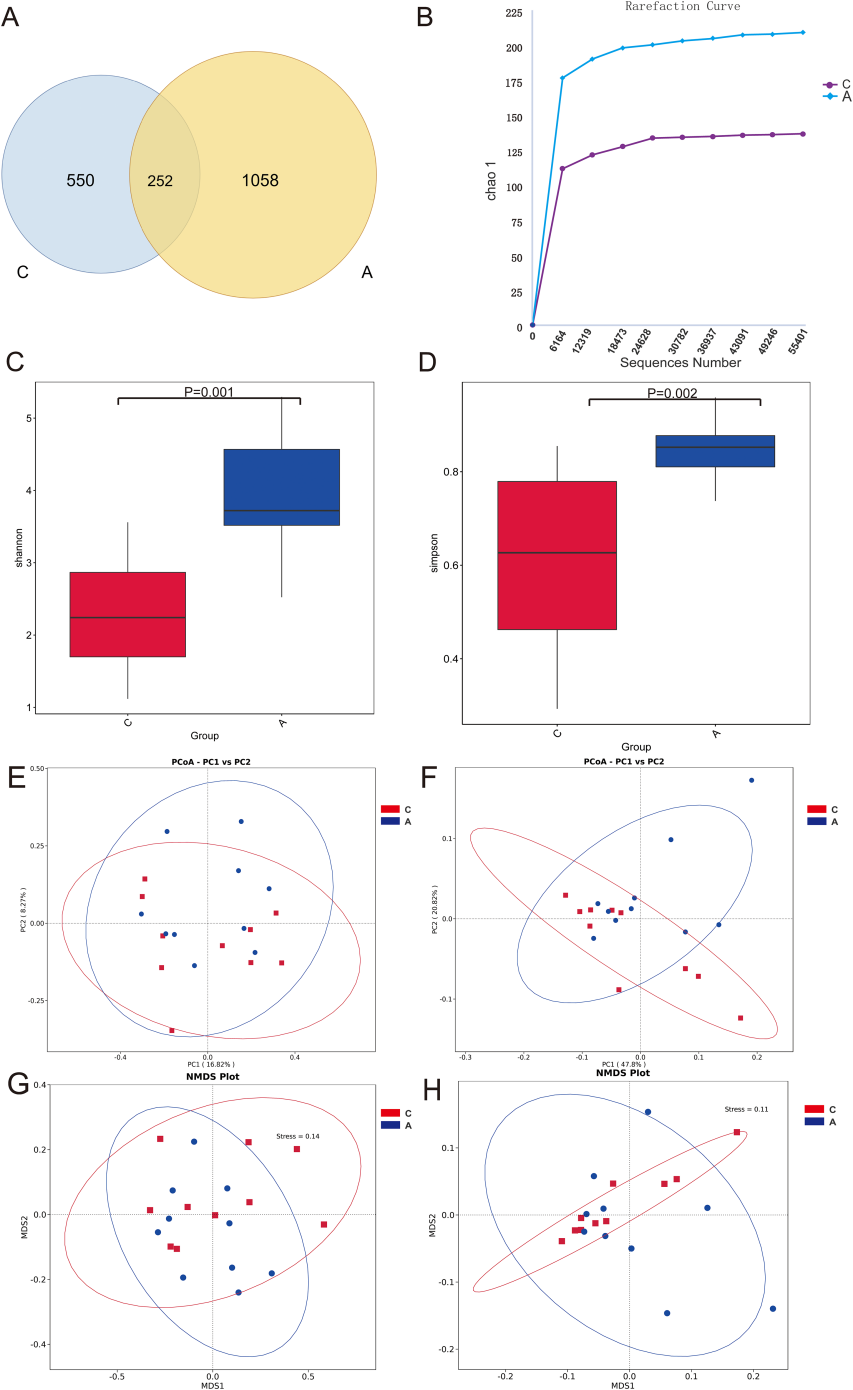
Microbiota diversity. **(A)** Venn diagram of the composition of ASVs in oral microbiota; **(B)** Alpha diversity rarefaction curves (Chao1 metric) for samples in different groups; **(C)** Wilcoxon rank-sum test for differences in Alpha diversity (Shannon index) between groups; **(D)** Wilcoxon rank-sum test for differences in Alpha diversity (Simpson index) between groups; **(E)** Beta diversity PCoA plots of unweighted UniFrac distance; **(F)** Beta diversity PCoA plots of weighted UniFrac distance; **(G)** Unweighted UniFrac distance NMDS plot; **(H)** Weighted UniFrac distance NMDS plot.

The analysis of oral microbiota structure between two groups revealed that the C group exhibited 550 unique ASVs, whereas the A group displayed 1058 unique ASVs, with 252 ASVs shared between the groups (**Figure 2A**). The analysis of species richness curves was conducted to assess the adequate representation of ASVs derived from Illumina MiSeq sequencing data. The 16S rRNA gene rarefaction curves for both study groups reached a plateau phase, signifying that the sequencing depth had achieved saturation, thereby ensuring that the sample coverage was comprehensive (**Figure 2B**). Samples were normalized to 55,401 sequences through rigorous standardization. Both the Shannon (Group C: [2.319], Group A: [3.908], P=0.001) and Simpson (Group C: [0.610], Group A: [0.844], P=0.002) indices, key alpha diversity metrics, demonstrated a statistically significant increase in Group A compared to Group C. These results were obtained using Wilcoxon rank sum test. These findings suggest that alcohol exposure may enhance the abundance and diversity of the oral bacterial communities (**Figures 2C, D**).

**Figure 3:**
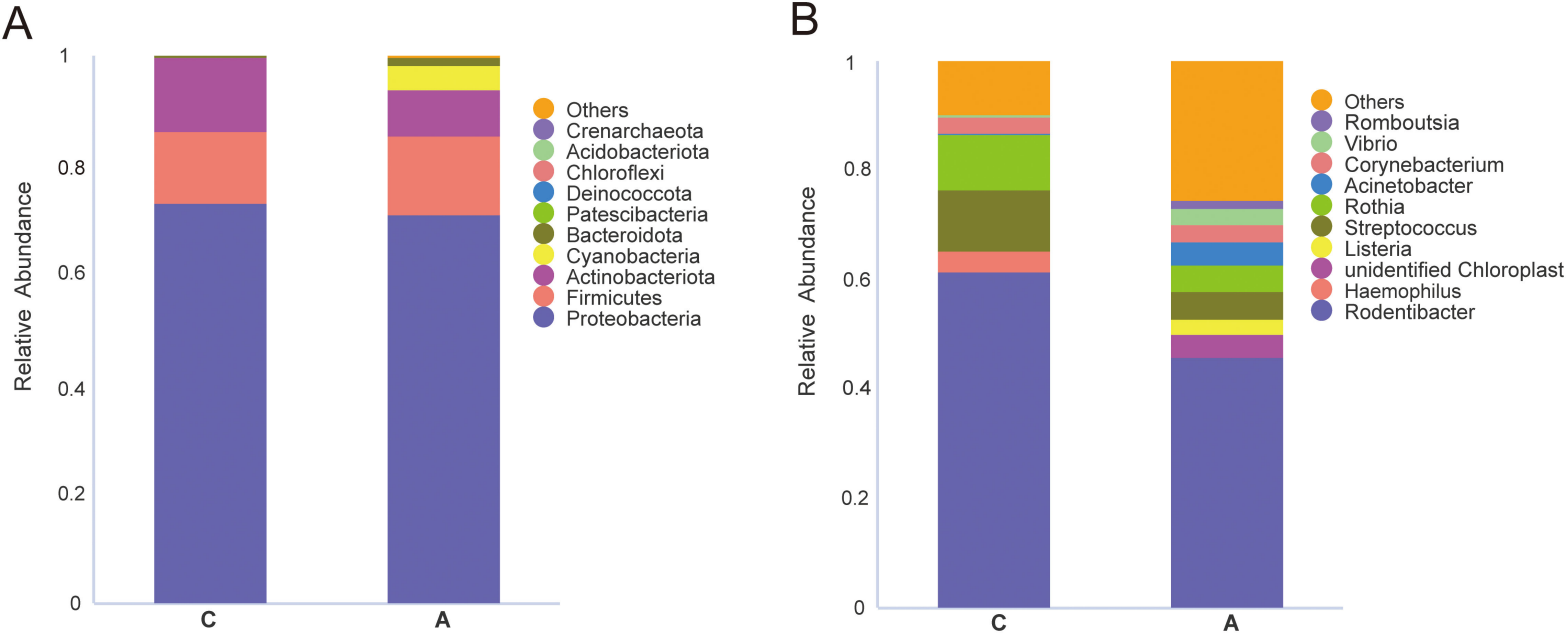
Relative abundance of the top 10 species at the level of phylum and genus. **(A)** phylum; **(B)** genus.

The beta diversity PCoA analysis, utilizing both weighted and unweighted UniFrac metrics, revealed pronounced disparities in microbial compositions between the groups (**Figures 2E, F**). Analysis of microbial community structure was visualized using NMDS plots. And distinct clustering was observed by the alcohol exposure (**Figures 2G, H**). Anosim and Adonis analyses were also used to compare dissimilarity between groups, which indicated significant differences (**Table 1**). Next, the relative abundances of bacterial phyla and genera were plotted for the top 10 most numerous species (**Figure 3**).

**Table 1:** Analysis of beta diversity of oral microbiota by Anonism and Adonis test.

Group	Anosim		Adonis	
	R	*P*-value	R^2^	Pr(>F)
C-A	0.300	0.005**	0.179	0.003**

** indicates significance compared with the C group by P<0.01.

**Figure 4:**
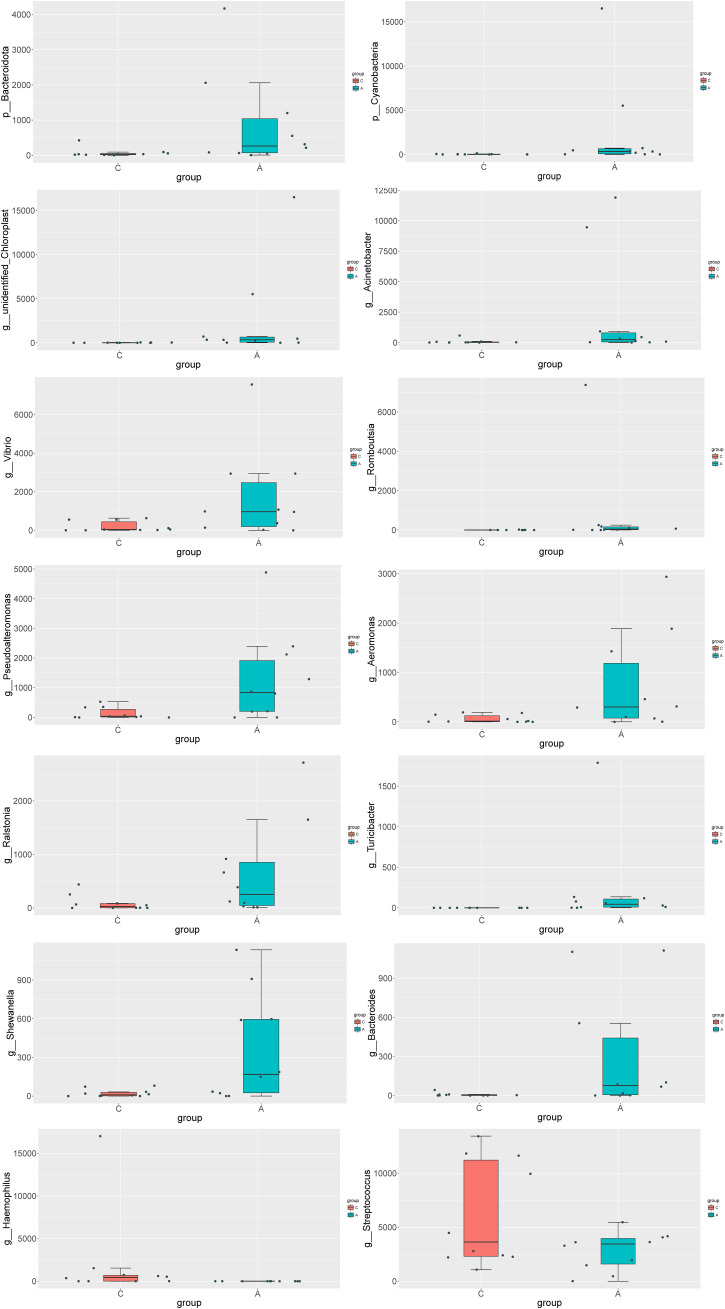
Analysis of the differences in species composition between groups at the Phylum level (p) and the Genus level (g).

MetaStat analysis, which further dissected the compositional differences in microbial communities between samples, revealed that the relative abundance of *Bacteroidota* and *Cyanobacteria* was significantly elevated in the A group at the phylum level. At the genus level, the relative abundance of several genera was significantly higher in the A group compared to the C group, including *unidentified_Chloroplast, Acinetobacter, Vibrio, Romboutsia, Pseudoalteromonas, Aeromonas, Ralstonia, Turicibacter, Shewanella*, and *Bacteroides*. In contrast, the genera *Haemophilus* and *Streptococcus* exhibited a significantly decreased abundance (**Figure 4**).

**Figure 5:**
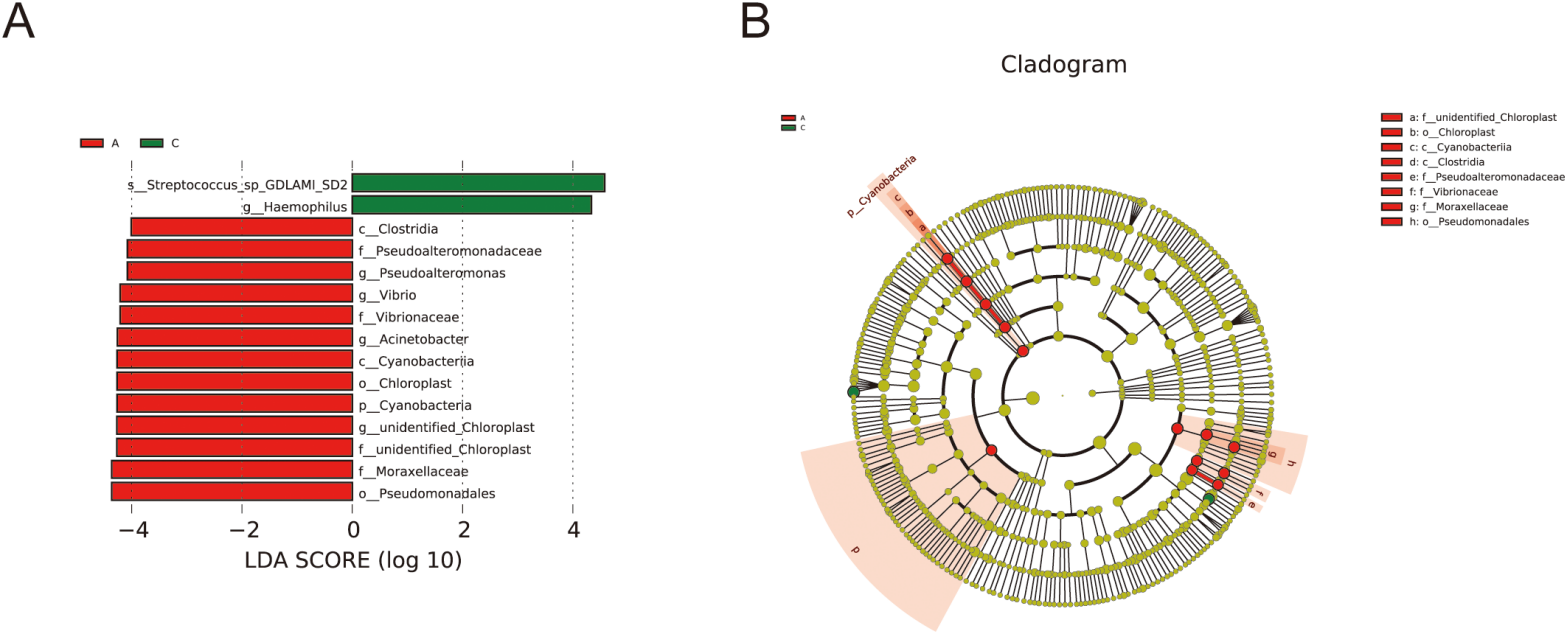
Analysis of species differences by LEfSe. **(A)** LDA diagram of LEfSe analysis, the absolute value of LDA was set at 4.0; **(B)** the cladogram shows phylogenetic distributions of oral bacteria between groups.

LEfSe analysis was performed to further delineate the differences in bacterial community structures (**Figure 5**). The analysis revealed significant increases in relative abundance of *Cyanobacteria, Cyanobacteriia, Chloroplast, unidentified_Chloroplast, Pseudomonadales, Moraxellaceae, Acinetobacter, Pseudoalteromonadaceae, Pseudoalteromonas, Vibrionaceae*, and *Vibrio* in the A group compared to the C group. Conversely, the relative abundance of *Haemophilus* and *Streptococcus_sp_GDLAMI_SD2* was significantly reduced.

#### 3.2.2 Correlation between oral microbiota and predictive functional pathways

**Figure 6:**
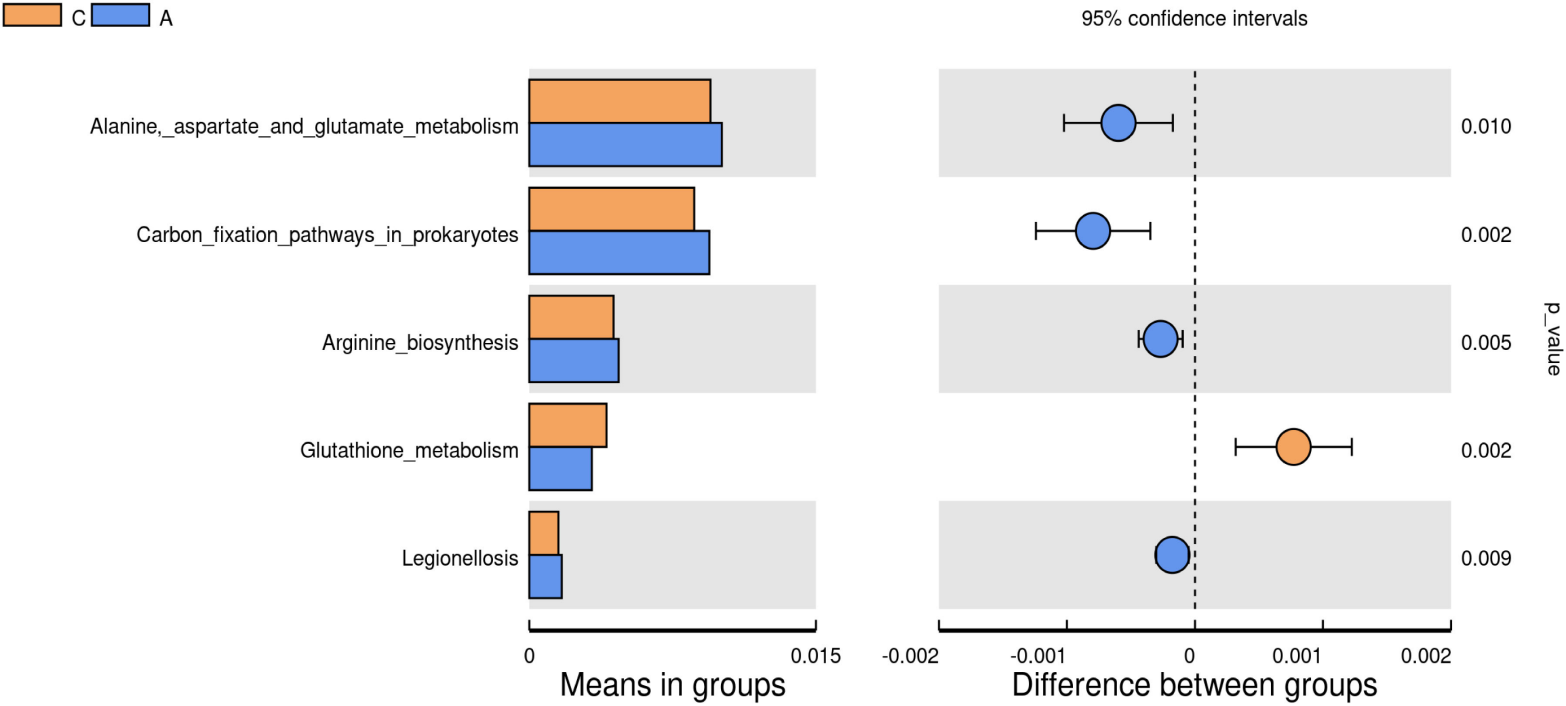
Analysis of the differences in metabolic functions between the groups (P<0.01).

Tax4Fun analysis of the 16S rRNA gene sequencing data was conducted to elucidate the functional trends within the oral microbiota. Metabolic functions associated with Alanine-aspartate and glutamate metabolism, carbon fixation pathways in prokaryotes, arginine biosynthesis, and Legionellosis were markedly elevated in the A group compared to the C group. In contrast, the metabolic function of glutathione metabolism was significantly reduced in the A group (P<0.01) (**Figure 6**).

### 3.3 Microbial co-occurrence network analysis

**Figure 7:**
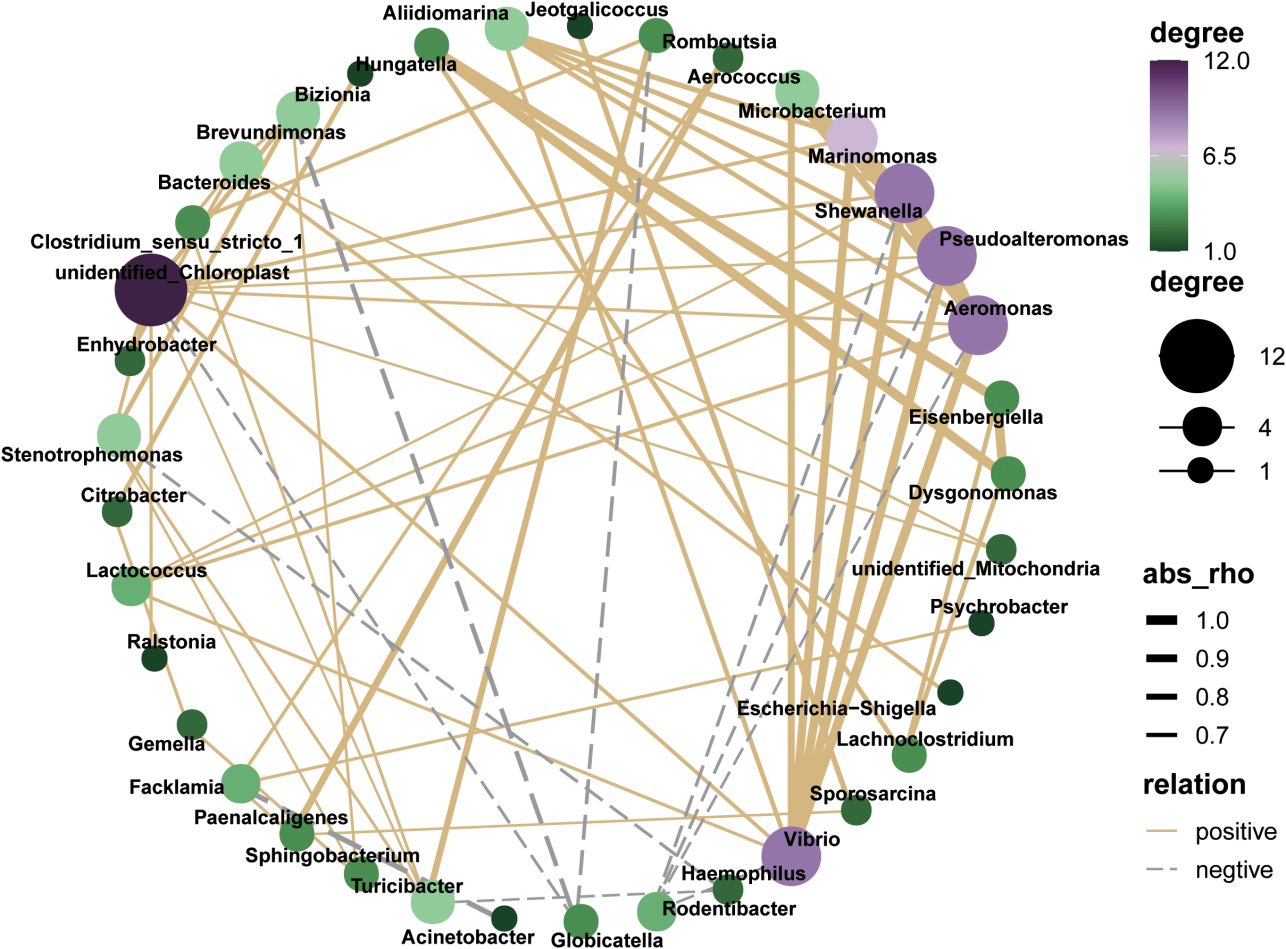
Co-occurrence network analysis of oral microbial genus based on Spearman’s correlation analysis (|R|> 0.6 and P < 0.05).

The interaction network of the microbial co-occurrence is plotted as follows (**Figure 7**). *Unidentified_Chloroplast, Vibrio, Pseudoalteromonadaceae, Aeromonas*, and *Shewanella* demonstrated markedly positive correlations with numerous hub genera, whereas *Rodentibacrer, Haemophilus* and *Globicatella* exhibited contrary patterns, predominantly displaying co-exclusion relationships.

## 4 Discussion

The oral microbiota is pivotal in both the initiation and advancement of oral diseases. Disruption of this balance, leading to dysbiosis and physiological alterations, can be detrimental to the host. Our study employed high-throughput 16S rRNA gene sequencing on oral swabs, uncovering the specific effects of alcohol exposure on oral microbiota diversity, composition, and functionality. This study is crucial for elucidating how alcohol modifies the oral ecosystem and subsequently impacts host health.

We observed significantly higher alpha diversity indices—Shannon and Simpson—in the A group compared to the C group. Consistent with our results, a large-scale population study by Fan et al. reported greater alpha diversity in drinkers’ oral microbiota, as measured by the Simpson index (Fan et al., 2018). While high species richness in the gut is typically indicative of a healthy microbiome (Lin et al., 2020), in the oral cavity, it is often linked to adverse outcomes, including poor oral health (Browning et al., 2024). This divergence might stem from alcohol’s modification of the oral host-environment dynamics, permitting increased bacterial species survival. Physiologically, alcohol exposure can alter the oral microbiota structure by impairing the phagocytic capacity of neutrophils, critical for antibacterial defense. Neutrophil dysfunction may foster abnormal oral bacterial proliferation and dissemination, increasing oral microbial diversity and, consequently, the risk of disease (Szabo, 1999).

Our study has revealed a notable increase in the abundance of *Bacteroidota* and *Bacteroides*. *Bacteroidota* are opportunistic pathogens, consisting of diverse Gram-negative bacteria capable of inducing the release of pro-inflammatory cytokines and initiating inflammatory responses (Yussof et al., 2020). Human infections with the *Bacteroides* result in a sequence of diseases, including meningitis, brain abscesses, oral infections, neck abscesses, lung abscesses, and inflammatory bowel diseases such as Crohn’s disease and intra-abdominal abscesses (Zafar and Saier, 2021).

The A group showed a significant increase in *Romboutsia* abundance, a genus positively associated with stage III periodontitis through gut microbiota analysis (Kawamoto et al., 2021). This suggests that potential oral-gut microbial crosstalk in alcohol-aggravated periodontal pathogenesis. Emerging evidence suggests that chronic alcohol consumption significantly elevate the relative abundance of *Romboutsia* within the rat oral microbiota, potentially exacerbating periodontitis progression through enhanced inflammatory responses and tissue degradation (Zhao et al., 2024).

*Acinetobacter* was more abundant in the A group. *Acinetobacter*, typically found in the gastrointestinal, respiratory, dermal, and urogenital tracts, is an opportunistic pathogen capable of inducing pneumonia, endocarditis, sepsis, urinary tract infections, and dermatitis (Lima et al., 2020). Previous studies suggest that exposure to ethanol may increase Acinetobacter pathogenicity. Ethanol has been demonstrated to exacerbate *Acinetobacter baumannii*-associated pneumonia and systemic spread by suppressing neutrophil antimicrobial activity (Gandhi et al., 2014).

The relative abundances of *Pseudoalteromonadaceae* and *Pseudoalteromonas*, as well as *Vibrionaceae* and *Vibrio*, were significantly elevated. Pseudoalteromonadaceae levels have significantly increased in the oral cavity of young women with recurrent aphthous stomatitis (Zhu et al., 2021). Certain *Pseudoalteromonas* species are known to be potentially harmful, exhibiting the capacity to produce pufferfish toxins, lysosomal substances, and autotoxins, which result in pathogenicity, food spoilage, environmental pollution, and biofouling (Jv et al., 2022). *Vibrio* species are intrinsic components of the estuarine water microbiota and are prevalent as infectious bacteria in the human oral cavity, with various pathogenic strains. Notably, *Vibrio parahaemolyticus* is the most frequently encountered, while *Vibrio vulnificus* poses the greatest risk, being the most lethal cause of vibriosis (Gomez-Gil et al., 2014). These *Vibrio* species can induce severe illnesses in individuals with pre-existing chronic conditions or compromised immune systems, potentially progressing to sepsis and death (Bell and Bott, 2021).

Bacteria of the *Turicibacter* genus are known to modulate the host’s lipid and cholesterol levels by modulating bile acid composition. They facilitate alterations in bile acid composition through deconjugation and dehydroxylation reactions *in vitro*, consequently impacting the host’s lipid and cholesterol profiles (Caslin et al., 2021). While current research does not directly link *Turicibacter* to alcohol consumption, it is hypothesized that alcohol’s influence on gut microbiota composition and functionality could indirectly modulate the role of *Turicibacter* in the gut, which includes the capacity to regulate bile acids and lipid metabolism.

In the A Group, there was a significant enrichment of *Cyanobacteria, Cyanobacteriia, Chloroplast*, the family-level *unidentified_Chloroplast*, and the genus-level *unidentified_Chloroplast*. The majority of *Cyanobacteria* can synthesize harmful cyanotoxins, which pose a significant risk to the health of humans and animals (Buratti et al., 2017). Beta-N-methylamino-L-alanine (BMAA), a neurotoxin produced by cyanobacteria, can bioaccumulate in the human body through the food chain, leading to neurodegenerative effects (Nunes-Costa et al., 2020). BMAA interacts with the mucosal immune system and the enteric nervous system via the mitochondrial pathway (Ra et al., 2021), and it significantly diminishes oxidative phosphorylation, disrupts intracellular calcium balance, and enhances the generation of reactive oxygen species (ROS) (Beri et al., 2017). These mechanisms could potentially account for some of the neurological symptoms seen in individuals with Alzheimer’s disease.

The *Aeromonas, Ralstonia*, and *Shewanella*, commonly found in aquatic habitats, have become a focal point in medical research due to their propensity for human colonization and infection. *Aeromonas* species, which can be pathogenic to humans, predominantly cause gastrointestinal diseases, wound infections, and soft tissue infections, along with clinical presentations such as septicemia (Pessoa et al., 2022). Adapted to low-nutrient conditions, the *Ralstonia* genus has been implicated in severe hospital-acquired infections, including osteomyelitis and meningitis (Nasir et al., 2019). *Shewanella* species are capable of inducing a spectrum of clinical diseases, encompassing skin and soft tissue infections, invasive diseases like septicemia, liver and gallbladder afflictions, otitis media, and its complications (Yu et al., 2022).

Conversely, we observed a significant decrease in the abundance of *Haemophilus*, a common oral symbiont, aligning with prior research (Liao et al., 2022). Such a reduction in *Haemophilus* has been previously documented in individuals with diabetes (Letchumanan et al., 2022) and those with oral squamous cell carcinoma (Al-Hebshi et al., 2017). Notably, *in vitro* studies have indicated that *Haemophilus paraphrophilus* exhibits anti-cancer properties (Baraniya et al., 2020). Additionally, we noted a significant decrease in the relative abundance of *Streptococcus* sp. *GDLAMI_SD2* is an important pathogen and host symbiont within the *Streptococcus* genus. However, the specific role of this strain remains elusive based on current research.

Our analysis of bacterial functional changes, informed by the KEGG database, revealed significant shifts in metabolic pathways in the A group. The primary distinctions between non-drinkers and drinkers were observed in metabolic functions, suggesting a potential link to the sustenance of oral homeostasis (Li et al., 2022). We found a significant increase in the metabolic function associated with “*Legionellosis*” in the A group. *Legionellosis*, a severe pneumonia caused by *Legionella* bacteria, can exceptionally lead to extrapulmonary infections such as those affecting the heart or wounds (Gonçalves et al., 2021). Furthermore, the metabolism of glutathione was significantly diminished in the A group, a finding consistent with previous studies. Glutathione, a crucial intracellular antioxidant, is known for its ability to neutralize free radicals and safeguard cellular integrity (Gasmi et al., 2024). In the co-occurrence network analysis, *Pseudoalteromonas* and *Aeromonas* were identified as hubs with high connectivity and high flora abundance. These genera exhibited significant correlations with a variety of other, suggesting their potential to serve as central nodes that integrate the oral microbiome. This role as integrators is crucial for mediating interactions among diverse microbial populations within the oral environment.

Our study has shown that alcohol consumption can lead to a decrease in the abundance of certain beneficial bacteria such as *Haemophilus*, while increasing the abundance of potentially pathogenic like *Bacteroides* and *Romboutsia*. This dysbiosis in the oral microbiome may contribute to the development of oral diseases such as periodontitis and dental caries. Furthermore, long-term alcohol exposure can have multifaceted negative impacts on oral health. It may impair saliva’s antimicrobial properties and host defense mechanisms. Both acute and chronic alcohol exposure can lead to functional changes in saliva, including decreased flow rate and impaired output of total protein, amylase, and electrolytes (Maier et al., 1986; Enberg et al., 2001). These alterations in saliva’s composition and function can further exacerbate the negative effects on oral health. Emerging evidence highlights the pivotal role of oral-gut axis dysbiosis in alcohol-related systemic pathologies. Pan et al. revealed that alcohol consumption may impacted systemic health through bacterial translocation along the oral-gut axis, which accelerates the progression of alcohol-related liver disease (ALD) (Pan et al., 2023).

Chronic alcohol consumption profoundly disrupts the composition and diversity of the gut microbiota. It leads to a reduction in beneficial commensal bacteria such as Bacteroidetes and Firmicutes, alongside increases in Gram-negative Proteobacteria and Gram-positive Actinobacteria (Li et al., 2024). These changes are associated with increased intestinal permeability, endotoxemia, and inflammation, which can exacerbate the progression of liver disease, cardiovascular disease, and other systemic conditions. Additionally, alcohol-induced gut dysbiosis can result in the overproduction of harmful metabolites such as lipopolysaccharides (LPS), which can enter the bloodstream and cause systemic inflammation (Xiang et al., 2024).

Future research should focus on elucidating the specific mechanisms by which alcohol alters human flora and the subsequent health implications. Additionally, longitudinal studies are needed to better understand the long-term consequences of alcohol consumption on both oral and systemic flora.

This study faces some limitations. The potential presence of contaminants represents a limitation of this study. The ASVs annotated as *Vibrio*, *Pseudoalteromonas, Aeromonas*, and chloroplast/cyanobacteria sequences are atypical for mammalian oral microbiomes and may originate from environmental contamination during sample processing such as reagents and laboratory surfaces or sequencing workflows, as previously reported in studies (Salter et al., 2014). Chloroplast sequences could also derive from plant material in the rats’ diet. Fortunately, their similar relative abundances in both groups may have reduced bias in inter-group comparisons. Future studies incorporating negative controls and contamination-aware bioinformatic pipelines such as *decontam* are warranted to refine the study. In addition, the shallow periodontal pockets in rats precluded the collection of subgingival microbiome samples alongside oral swab samples. Consequently, while our oral swab samples yielded a diverse microbial collection, this may not represent the subgingival plaque community. Future research should aim to collect subgingival samples to garner a more precise understanding of microbial communities. Additionally, future studies should delve into the metabolomics of the oral microbiota to elucidate the mechanisms by which specific bacterial metabolic byproducts either foster or hinder disease progression.

## 5 Conclusion

In conclusion, chronic alcohol exposure is associated with a substantial reconfiguration of the microbial community, which in turn can influence the host’s metabolic and immunological profiles. Our findings offer novel insights into the complex interplay between alcohol intake and the microbiome. By characterizing these microbial shifts, our study underscores the critical role of alcohol cessation in enhancing both oral and systemic health. Subsequent research should aim to clarify the mechanisms by which these microbial alterations impact host health via their metabolic byproducts, potentially unveiling new therapeutic targets for the mitigation of alcohol-associated diseases.

## Data Availability

The datasets presented in this study can be found in online repositories. The names of the repository/repositories and accession number(s) can be found in the article/supplementary material.
